# Analysis of Hyperosmotic Tolerance Mechanisms in *Gracilariopsis lemaneiformis* Based on Weighted Co-Expression Network Analysis

**DOI:** 10.3390/genes15060781

**Published:** 2024-06-13

**Authors:** Baoheng Xiao, Xiaoqing Feng, Pingping Li, Zhenghong Sui

**Affiliations:** Key Laboratory of Marine Genetics and Breeding, Ocean University of China, Ministry of Education, Qingdao 266003, China; klad1990@163.com (B.X.); 17086260662@163.com (X.F.); lpp0780@163.com (P.L.)

**Keywords:** *Gracilariopsis lemaneiformis*, high salinity, modules, WGCNA

## Abstract

We conducted transcriptome sequencing on salt-tolerant mutants X5 and X3, and a control (Ctr) strain of *Gracilariopsis lemaneiformis* after treatment with artificial seawater at varying salinities (30‰, 45‰, and 60‰) for 3 weeks. Differentially expressed genes were identified and a weighted co-expression network analysis was conducted. The blue, red, and tan modules were most closely associated with salinity, while the black, cyan, light cyan, and yellow modules showed a close correlation with strain attributes. KEGG enrichment of genes from the aforementioned modules revealed that the key enrichment pathways for salinity attributes included the proteasome and carbon fixation in photosynthesis, whereas the key pathways for strain attributes consisted of lipid metabolism, oxidative phosphorylation, soluble N-ethylmaleimide-sensitive factor-activating protein receptor (SNARE) interactions in vesicular transport, and porphyrin and chlorophyll metabolism. Gene expression for the proteasome and carbon fixation in photosynthesis was higher in all strains at 60‰. In addition, gene expression in the proteasome pathway was higher in the X5-60 than Ctr-60 and X3-60. Based on the above data and relevant literature, we speculated that mutant X5 likely copes with high salt stress by upregulating genes related to lysosome and carbon fixation in photosynthesis. The proteasome may be reset to adjust the organism’s proteome composition to adapt to high-salt environments, while carbon fixation may aid in maintaining material and energy metabolism for normal life activities by enhancing carbon dioxide uptake via photosynthesis. The differences between the X5-30 and Ctr-30 expression of genes involved in the synthesis of secondary metabolites, oxidative phosphorylation, and SNARE interactions in vesicular transport suggested that the X5-30 may differ from Ctr-30 in lipid metabolism, energy metabolism, and vesicular transport. Finally, among the key pathways with good correlation with salinity and strain traits, the key genes with significant correlation with salinity and strain traits were identified by correlation analysis.

## 1. Introduction

*Gracilariopsis lemaneiformis* is an important macroalga in the phylum Rhodophyta. It has various applications, such as improving the ecological environment and serving as bait for abalone and as a raw material for agar extraction [1,2,3,4]. To develop a strain with a high agar content, we used a high osmotic screening (high-salinity) treatment to screen for mutated tetraspores. After three consecutive high-salinity screenings, we obtained mutants X3 and X5 that tolerate high salinity, where X5 exhibited a higher agar content than the other mutants when cultured at a normal salinity, while the agar content of X3 was almost indistinguishable from the control [5].

We used high osmotic pressure to screen for high agar content in *G. lemaneiformis* for several reasons. First, when plants experience high osmotic pressure stress, their response typically includes osmotic regulation, the removal of reactive oxygen substances, and the thickening of cell walls [6,7,8,9,10]. To balance internal and external osmotic pressures, cells produce osmolytes, which mainly consist of polyols (e.g., mannitol, sorbitol, and xylitol), amino acid analogs (e.g., betaine, proline, and ectoine), lipids (e.g., glycerol), and soluble sugars and their derivatives [11,12,13]. In response to oxidative stress, plant cells produce various enzymes that reduce cell damage. Superoxide dismutase, catalase, glutathione peroxidase, and glutathione reductase help remove oxidative species [14,15]. In addition, cells produce major antioxidants such as glutathione, vitamins (vitamin C), and pigments (carotenoids) to further clear oxidative substances, thereby minimizing oxidative damage caused by osmotic stress [16,17]. Cells also respond to osmotic stress-induced cell deformation by thickening the structure of the cell wall to maintain a normal cell shape [18,19,20]. Therefore, individuals with mutations that result in increased intracellular solute content and thickened cell walls are more likely to survive in environments with high osmotic pressure. Because variant X5 has a high agar content and a thick cell wall, it can more readily cope with high salt stress compared to other strains. However, it is not yet clear what the other factors, key pathways, and key genes involved in the tolerance of strains X5 and X3 to high salt environments are.

Moreover, *G. lemaneiformis* grows in the intertidal zone, and the mechanisms of its adaptation to salinity change (hypersalinity) are unclear. Therefore, the goal of this study from one aspect was to identify the key pathways and genes involved in the response of *G. lemaneiformis* variants to a high-salinity environment by analyzing the key pathways and genes that are co-varied between mutants and controls in response to high salt stress. From another aspect, by analyzing the key pathways and genes that differentiate the mutant from the control, we were able to resolve the reasons why the mutant was more tolerant to high salt stress compared to the control. We analyzed the transcriptomic data from mutants that tolerate high salinity and control under high salt stress using weighted correlation network analysis (WGCNA). Our results provide a reference for further studies on salt tolerance in algae, and they can be applied in the development of cultivars with a high agar content.

## 2. Materials and Methods

### 2.1. Experimental Materials

The high-salinity-tolerant mutants X3 and X5 were maintained by the Key Laboratory of Marine Genetics and Breeding Ministry of Education (Ocean University of China). These mutant (X3, X5) strains were obtained by the mutagenesis of haploid spores, and these spores are released by WLP-1. We used a haploid strain that the haploid individual obtained from spores produced by the laboratory-preserved WLP-1 strain [5] as the control (Ctr). Strain X5 had stronger salt tolerance qualities than X3, and strain X3 had stronger salt tolerance traits than the control [5].

### 2.2. Material Handling and Sampling

Mutants X5 and X3 and the control were cultivated in normal seawater supplemented with Pro media [21] at a temperature of 20 °C, light intensity of 30 μmol m^−2^ s^−1^, and a light–dark cycle of 12 L:12 D. The seawater was replaced every 3 days. 

After accumulating a certain amount of biomass, 0.2–0.3 mg of each strain was sampled and used to set up three replicates for each group, which were incubated in artificial seawater [22] with salinities of 30‰, 45‰, and 60‰ to create groups X5-30, X5-45, X5-60, X3-30, X3-45, X3-60, Ctr-30, Ctr-45, and Ctr-60. The samples were incubated at a temperature of 20 °C, light intensity of 15 μmol m^−2^ s^−1^, and a light–dark cycle of 12 L:12 D. The artificial seawater was changed once every 7 days. The samples were collected after 3 weeks for subsequent analysis.

### 2.3. Total RNA Extraction and Library Construction

Total RNA was extracted using the Tenge Polysaccharide Polyphenol Kit (QIAGEN, Hilden, Germany), and tested for quality using an Agilent 2100 Bioanalyzer (Agilent, Santa Clara, CA, USA). We used Oligo(dT) magnetic beads to enrich the mRNA containing polyA tails. The NEBNext^®^ Ultra™ RNA Library Prep Kit for Illumina^®^ (NEB, Ipswich, MA, USA) was used to create libraries from mRNAs. The library was sequenced using the Illumina NovaSeq 6000 (San Diego, CA, USA) platform. The obtained mRNA was randomly fragmented using a divalent cation in the NEB Fragmentation Buffer. The fragmented mRNA was then used as a template, and random oligonucleotides were used as primers to synthesize the first strand of cDNA in the presence of M-MuLV reverse transcriptase. The RNA chain was then degraded using RNaseH, and the second strand of cDNA was synthesized using DNA polymerase I system. The purified double-stranded cDNA was subjected to end repair, A-tailing, and sequencing adapter ligation. cDNA fragments of approximately 250–300 bp were selected using AMPure XP beads, followed by PCR amplification and purification of the PCR products using AMPure XP beads. Finally, the library was obtained. Library construction and sequencing were performed by Suzhou PANOMIX Biomedical Tech Co., Ltd. (Suzhou, China). 

### 2.4. Quality Control and Analysis of Sequencing Data

The raw data obtained after sequencing were filtered, the sequencing error rate was evaluated, and the GC content distribution was validated to obtain clean reads for further analysis. After quality control, the clean reads were compared to the reference genome of *G. lemaneiformis* (no. SRR20338037) for assembly, and the HISAT2 (Version 2.0.5) software [23] was used to compare the clean reads with the reference genome to acquire read localization information. We then conducted quantitative analysis, differential gene analysis, and enrichment analysis. Quantitative analysis was conducted using software featureCounts (Version 1.5.0-p3) and stringtie (Version 1.3.3b). Differential gene analysis was performed using software DESeq2 (Version 1.16.1) and edgeR (Version 3.18.1). The differential gene sets were subjected to GO and KEGG enrichment analysis using the clusterProfile (Version 3.4.4) software, with a significance enrichment threshold of Padj < 0.05 for both GO functional enrichment and KEGG enrichment analysis. Table S1 (Supplementary Materials) listed the number of reads, sequencing error rate, GC content distribution, and comparison rate after the raw data were filtered.

### 2.5. Weighted Correlation Network Analysis (WGCNA)

WGCNA analysis was performed using the R language package (version 3.5.0) (https://cran.r-project.org/, accessed on 10 November 2021). After importing the preprocessed expression scale, the expression matrix was created by filtering the data using the goodsamplegenes (gsg) function and clustering the samples using flashclust. The cor function, pheatmap package, and clusterProfiler package were then used for correlation analysis, cluster heatmap analysis and enrichment analysis, etc. [24].

## 3. Results

### 3.1. Correlation Analysis of Samples and Traits

**Figure 1:**
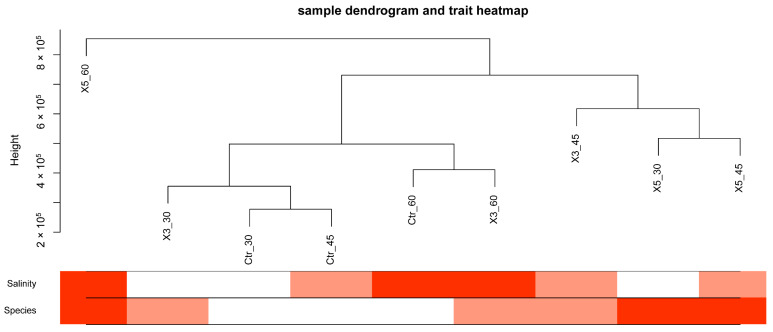
Demonstration of the correlation between samples and traits. Note: The darker the color, the stronger the correlation.

Figure 1 shows a heatmap of the sample–trait association. The clustering was divided into three branches. The X5-60 sample was in a separate branch, which indicated that its gene expression pattern differed significantly from that of the other samples. Groups X5-30, X5-45, and X3-45 were clustered into one branch, with X5-30 and X5-45 clustering first among the three samples. This result suggested that the gene expression patterns of the X5 strain cultured at the salinities of 30‰ and 45‰ were very comparable. They then clustered with X3-45, which showed that the gene expression pattern of strain X3 at 45‰ was similar to that of strain X5 at 30‰ and 45‰. The last branch contained the remaining five samples. Ctr-30 and Ctr-45 clustered together, revealing similar gene expression patterns at the salinities of 30‰ and 45‰. The presence of X3-30 in this branch indicated comparable gene expression patterns to those of the control at the salinities of 30‰ and 45‰, but the pattern differed significantly at 45‰. The clustering of the Ctr-60 and X3-60 samples indicated that their gene expression patterns were more similar to each other than to that of X5-60, suggesting that Ctr-60 and X3-60 may have more similar physiological responses to high osmolarity. 

The graph in the lower section of Figure 1 shows that the samples with the strongest association with salinity were X5-60, X3-60, and Ctr-60, followed by X5-45, X3-45, Ctr-45, and finally X5-30, X3-30, and Ctr-30. The samples with the highest correlation with the strain were X5-60, X5-45, X5-30, and, to a lesser extent, X3-60, X3-45, and X3-30, followed by Ctr-60, Ctr-45, and Ctr-30.

### 3.2. Correlation Analysis between Samples, Traits, and Modules

**Figure 2:**
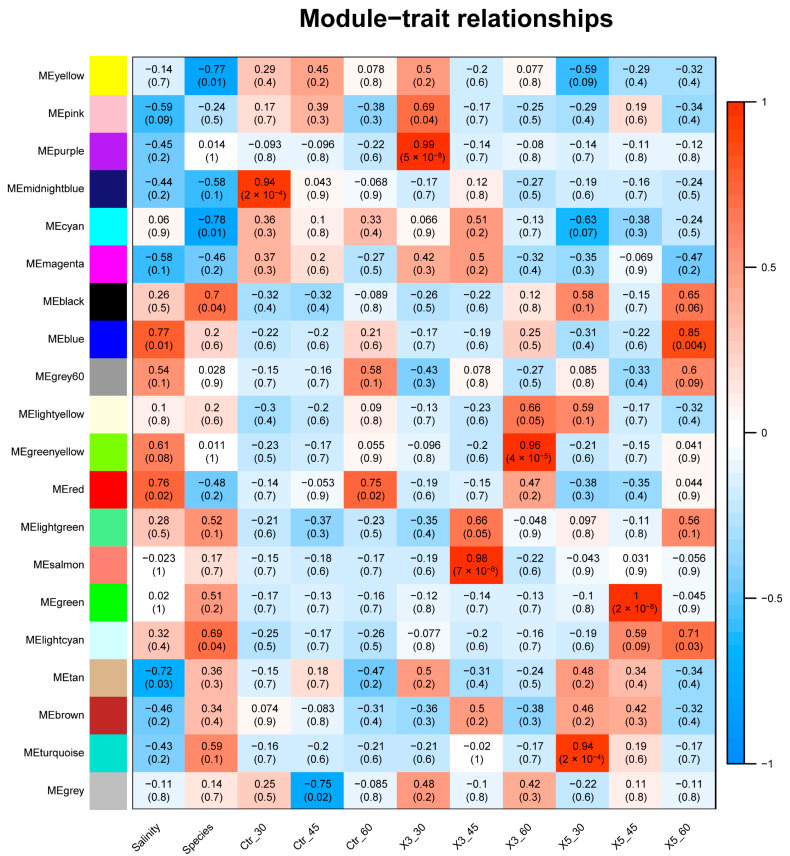
Heatmap of correlations between samples, traits, and modules.

The heatmap shows the correlation between the samples, traits, and color modules, where vertical coordinates represent the traits and 11 samples, and horizontal coordinates represent the various color modules (Figure 2). The number in each grid indicates the degree of correlation between the module and the sample or trait. An absolute correlation value close to one indicates a stronger positive correlation between the module and the sample or trait. The number in parentheses is the *p*-value. The blue, red, and tan modules show strong correlations with salinity features and significant *p*-values. The yellow, cyan, black, and light-cyan modules had the highest correlations to strain attributes and significant *p*-values.

### 3.3. Module Gene Expression Pattern Analysis and Enrichment Pathway Analysis

**Figure 3:**
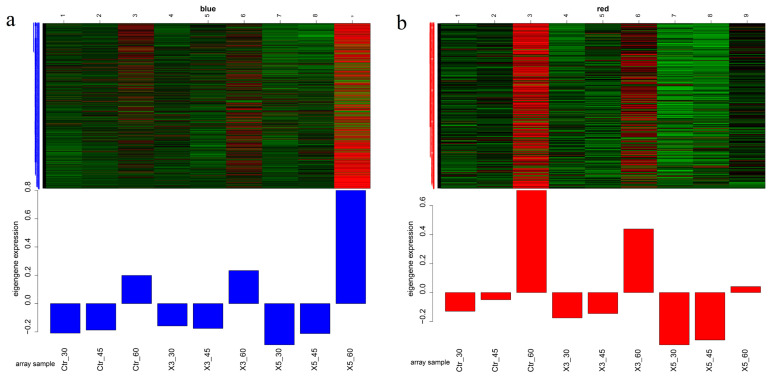
Modular gene expression patterns with a good correlation with salinity: (**a**) blue module; (**b**) red module.

The genes in both the blue and red salinity-related modules for all three strains showed an upward trend as salinity increased (Figure 3). At 60‰, the expression of genes in the blue module was higher in strain X5 than in the Ctr and X3 groups. The genes in this module may be critical genes for the tolerance of the X5 strain to high salinity. At 60‰, the expression of genes in the red module was higher in the Ctr group than in the X3 and X5 groups. The genes in this module may be important for the response of the control strain to extreme salt stress. These results indicate that the genes in both modules are related to how the three strains cope with high-salinity environments. 

**Figure 4:**
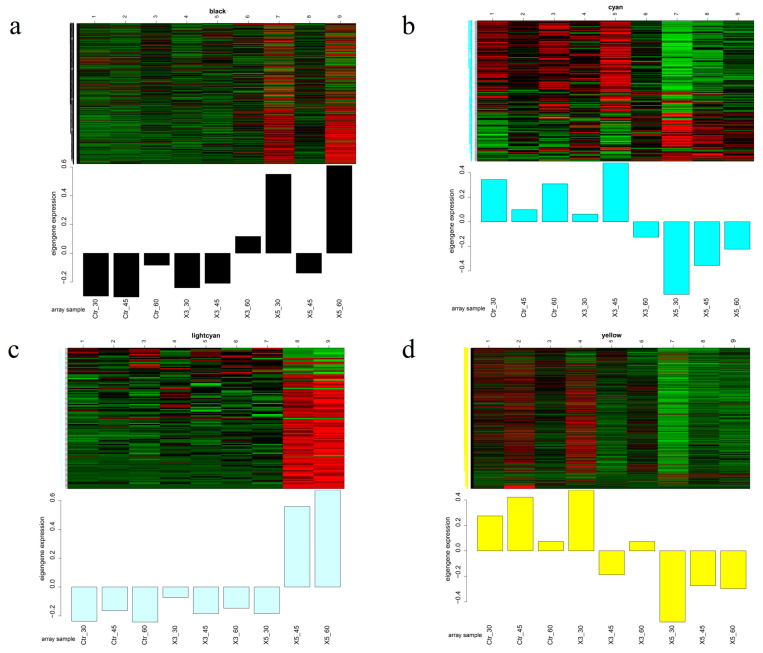
Modular gene expression patterns with a good correlation with the strains: (**a**) black; (**b**) cyan; (**c**) light cyan; (**d**) yellow.

Figure 4 depicts the modules that are closely related to the strain attributes. In the black module, at 30‰, the gene expression of both the Ctr and X3 samples was downregulated while that of strain X5 was upregulated. At 45‰, the gene expression of all three strains was downregulated. At the salinities of 30‰ and 60‰, expression of genes in the X5 strain was higher compared to those in the Ctr and X3 groups. For the cyan mode, genes in the X5 strain showed lower expression compared to those of the Ctr and X3 samples at 30‰, 45‰, and 60‰. In the light-cyan module, the gene expression of the X5 strain was higher than that of the Ctr and X3 groups at 45‰ and 60‰. In the yellow module at all three salinities, expression of genes in strain X5 was lower than that in the other two groups. These results suggest that the traits of the X5 strain differ from those of the Ctr and X3 strains.

**Figure 5:**
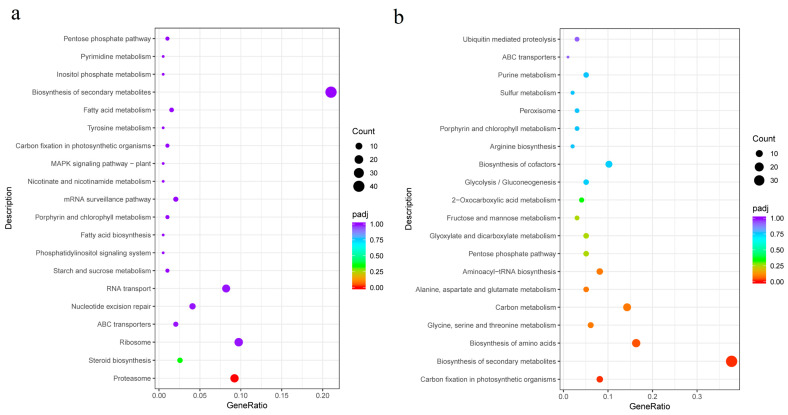
Scatter plot of enrichment pathways of genes in the blue and red modules. Note: The horizontal coordinate is the ratio of the number of differential genes annotated to the total number of differential genes in the KEGG enrichment pathway, and the vertical coordinate is the KEGG enrichment pathway. The size of the dots in the graph represents the number of genes annotated to the KEGG enrichment pathway, and the colors from red to purple represent the significance size of the enrichment. (**a**) Blue module; (**b**) red module.

The top 20 enriched metabolic pathways present in the blue and red modules based on KEGG enrichment analysis are shown in Figure 5. For the blue module, they include the proteasome, steroid biosynthesis, ribosome, and ABC transport pathways and others. Based on the q-value, only the proteasome pathway showed a significant difference. Moreover, the number of genes involved in the secondary metabolite synthesis pathway was the highest. In the red module, the significantly different enrichment pathways were the carbon fixation of photosynthetic organisms, biosynthesis of secondary metabolites, and amino acid biosynthesis. 

**Figure 6:**
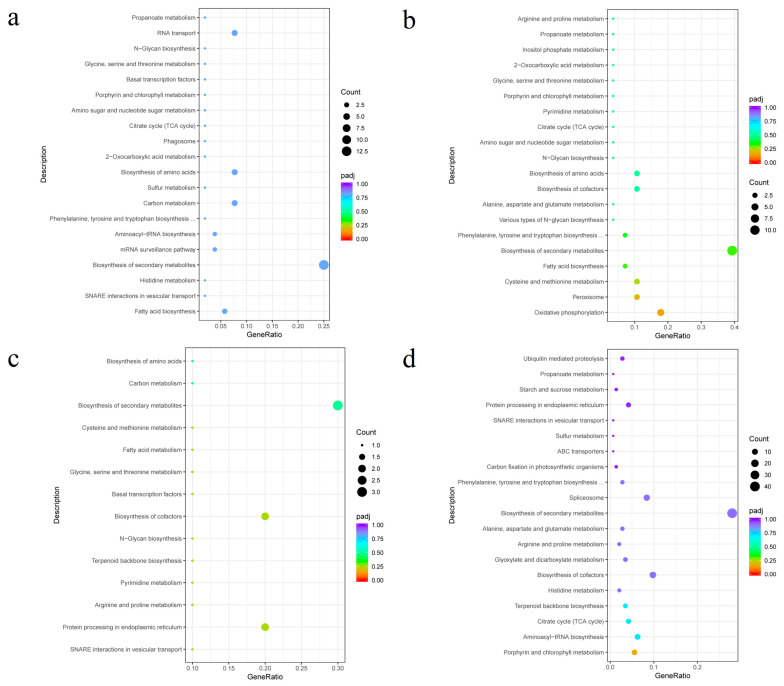
Scatter plot of enrichment pathways for genes in black, cyan, light-cyan, and yellow modules. Note: The horizontal coordinate is the ratio of the number of differential genes annotated to the total number of differential genes in the KEGG enrichment pathway, and the vertical coordinate is the KEGG enrichment pathway. The size of the dots in the graph represents the number of genes annotated to the KEGG enrichment pathway, and the colors from red to purple represent the significance of the enrichment: (**a**) black; (**b**) cyan; (**c**) light cyan; (**d**) yellow.

Figure 6 depicts the top 20 enriched metabolic pathways of the black, cyan, light-cyan, and yellow modules based on KEGG enrichment analysis. In the black module, they included fatty acid biosynthesis, soluble N-ethylmaleimide-sensitive factor-activating protein receptor (SNARE) interactions in vesicular transport, histidine metabolism, etc. The secondary metabolite synthesis pathway had the highest gene ratio. In the cyan module, the top 20 enriched pathways included oxidative phosphorylation, peroxisome, cysteine and methionine metabolism, etc. In the light-cyan module, these pathways included the interaction of SNARE in vesicle transport, protein processing in the endoplasmic reticulum, arginine and proline metabolism, and so on. The top 20 enriched pathways in the yellow module included porphyrin and chlorophyll metabolism, aminoacyl-tRNA biosynthesis, the tricarboxylic acid cycle, etc. However, the above enrichment pathways showed no significant difference.

### 3.4. Expression Analysis of Genes in the Most Significant Enrichment Pathway Correlated with Salinity and Strain

**Figure 7:**
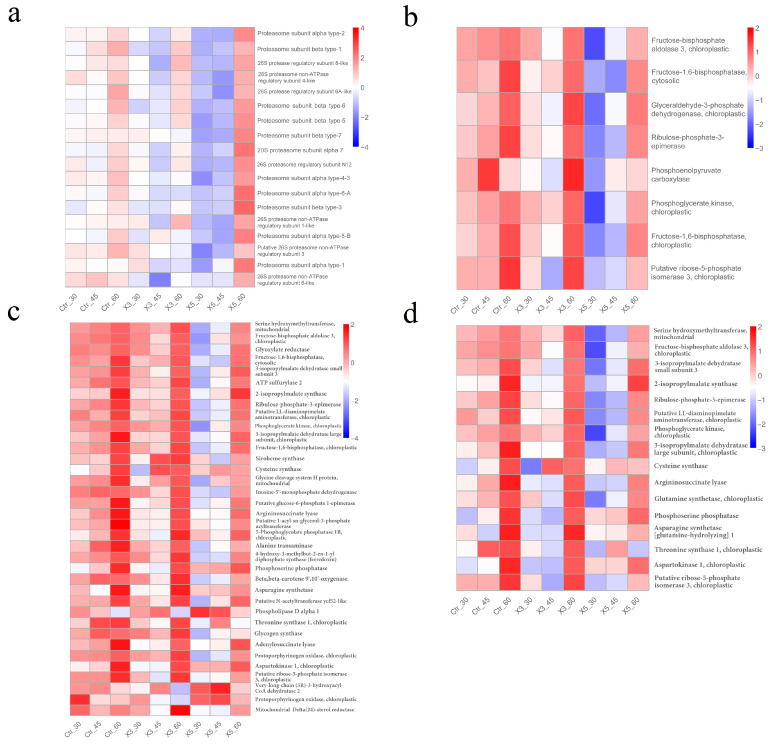
Analysis of gene expression in the main enrichment pathways of blue and red modules. Note: The vertical coordinates are the normalized values of the differential gene FPKM. (**a**) Genes in the pathway of proteasome; (**b**) carbon fixation by photosynthesis; (**c**) biosynthesis of secondary metabolites; and (**d**) biosynthesis of amino acids.

The genes in the enrichment pathways correlated with salinity were then examined in terms of expression in each sample. Figure 7 shows a heatmap of the expression of genes from the proteasome (blue), carbon fixation by photosynthesis (red), biosynthesis of secondary metabolites (red), and biosynthesis of amino acids (red), which were significantly different from the enrichment pathways in the blue and red module, respectively. Most of the genes were highly expressed in samples Ctr-60, X3-60, and X5-60, particularly in the latter, while all the genes were downregulated in the X5-30 and X5-45 groups (Figure 7a). At 60‰ salinity, the proteasome (Figure 7a) was highly active in all strains, particularly in X5-60. The proteasome is primarily responsible for protein degradation [25], and it has been suggested that cells may adapt to a high-salinity environment by modifying their proteomes through protein degradation. Most of the genes related to carbon fixation in photosynthesis were highly expressed in Ctr-60, X3-60, and X5-60 (Figure 7b), which suggests that carbon fixation in photosynthesis in all samples improved to adapt to a high salt environment. The expression of most of these genes was higher in Ctr-60 and X3-60 than in X5-60. In response to high salt stress (60‰), carbon fixation by photosynthesis in the Ctr-60 and X3-60 groups may be more active compared to that in X5-60. Similarly, most of the genes from the biosynthesis of secondary metabolites and biosynthesis of amino acids were highly expressed in Ctr-60, X3-60, and X5-60, and the expression of most of these genes was higher in Ctr-60 and X3-60 than in X5-60 (Figure 7c,d). The gene function of biosynthesis of secondary metabolites enrichment pathway involves amino acid metabolism, sugar metabolism, and lipid metabolism, while the biosynthesis of amino acids enrichment pathway involves the metabolism of threonine, glutamate, and aspartate. The differences in the gene expression between these two enrichment pathways in different strains at a salinity of 60‰ suggests that X5-60 differs from Ctr-60 and X3-60 in multiple metabolic pathways.

**Figure 8:**
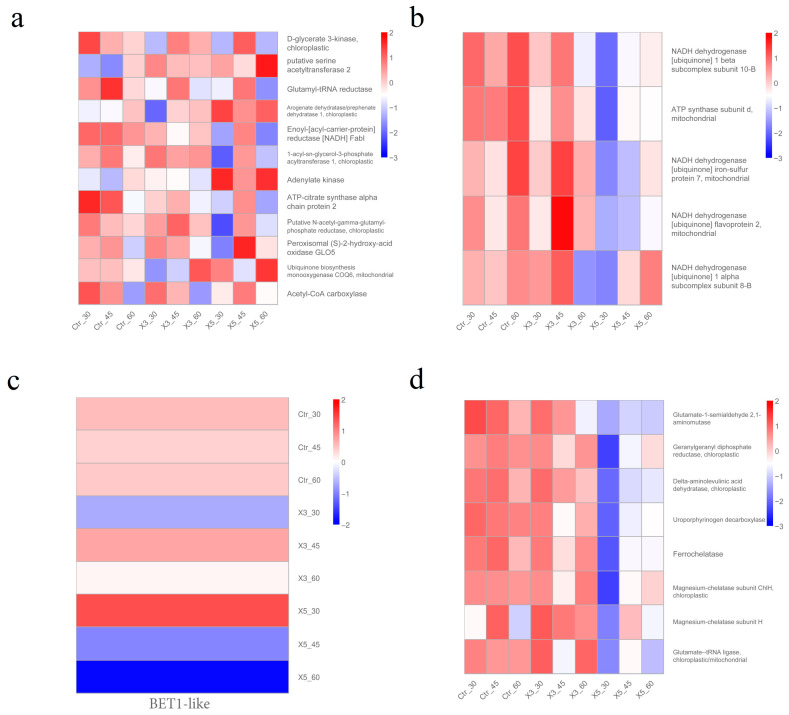
Analysis of gene expression in the main enrichment pathways of black (**a**), cyan (**b**), light-cyan (**c**) and yellow (**d**) modules. Note: The vertical coordinates are the normalized values of the differential gene FPKM. (**a**) genes in the pathway of the synthesis of secondary metabolites; (**b**) oxidative phosphorylation; (**c**) SNARE interactions in vesicular transport; and (**d**) porphyrin and chlorophyll metabolism.

Figure 8 depicts the heatmaps of the expression of genes from the most important enriched pathways: secondary metabolite synthesis (black), oxidative phosphorylation (cyan), SNARE interactions in vesicular transport (light cyan), and porphyrin and chlorophyll metabolism (yellow). At 30‰, the expression of genes in the X5 samples differed greatly from that of the control, with the exception of glutamyl-tRNA reductase, acetyl-CoA carboxylase, and ubiquinone biosynthesis monooxygenase genes (Figure 8a). Even if there were genes of which the expression was both up- and downregulated, their expression level would not be to the same degree. At 30‰ salinity, the expression in X5 samples of the genes involved in oxidative phosphorylation and porphyrin and chlorophyll metabolism was downregulated, while that in the Ctr and X3 groups was upregulated (Figure 8b,d). At 30‰ salinity, the expression in X5 samples of the genes involved in SNARE interactions in vesicle transport was upregulated relative to Ctr and X3 (Figure 8c). 

**Figure 9:**
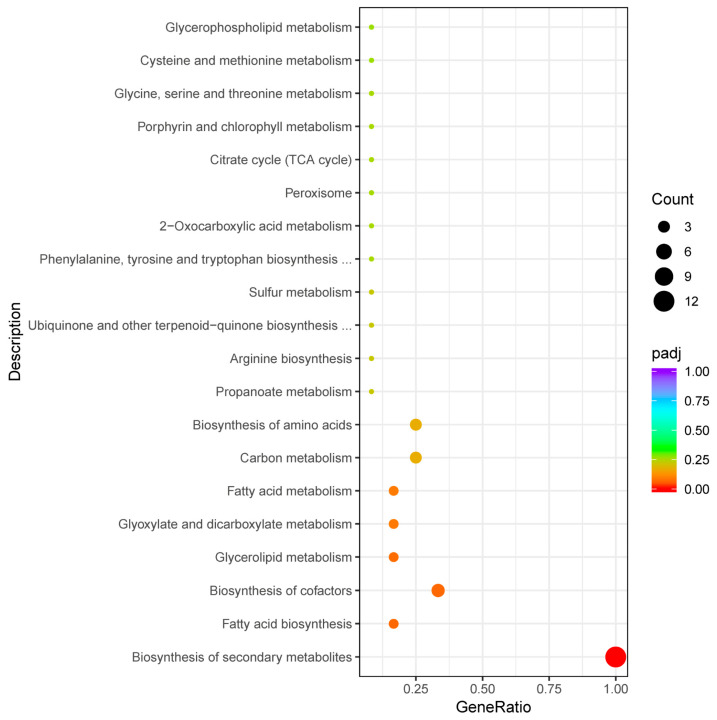
KEGG enrichment pathway for genes in the secondary metabolite enrichment pathway. Note: The horizontal coordinate is the ratio of the number of differential genes annotated to the total number of differential genes in the KEGG enrichment pathway, and the vertical coordinate is the KEGG enrichment pathway. The size of the dots in the graph represents the number of genes annotated to the KEGG enrichment pathway, and the colors from red to purple represent the significance of the enrichment.

Because the synthesis of secondary metabolites (Figure 8a) was not specific to an exact pathway, we subjected these genes to a second round of KEGG analysis. Figure 9 shows gene enrichment along the secondary metabolite route. In addition to the biosynthesis of secondary metabolites, the major enriched metabolic pathways included fatty acid biosynthesis, cofactor biosynthesis, glycerol ester metabolism, fatty acid metabolism, and so on. These results show that these different pathways were primarily related to lipids. In terms of strain characteristics, the X5-30 samples may have differed significantly from Ctr-30 and X3-30 samples in lipid metabolism (Figure 8a), oxidative phosphorylation metabolism (Figure 8b), SNARE interactions in the vesicular transporter (Figure 8c), and porphyrin and chlorophyll metabolism (Figure 8d). 

### 3.5. Association Analysis of Genes and Traits

The WGCNA analysis identified associations between all genes and traits (salinity and strain), which allowed us to screen for genes that were in the most differentially enriched pathway and also had a strong correlation with the trait after setting a threshold value (i.e., the target gene that was in the most differentially enriched pathway and had a *p*-value < 0.05). We then chose candidate genes from the proteasome and carbon fixation pathways of photosynthesis that showed a strong connection with salinity characteristics and had a *p* value < 0.05. Table 1 provides the basic information about the genes which had correlation values ranging from 0.69 to 0.78. Five of the genes associated with salinity features were involved in carbon fixation via photosynthesis, and two were involved in the proteasome metabolic pathway. These seven genes may be key to conferring high salinity tolerance in *G. lemaneiformis*. Genes related to carbon fixation in photosynthesis are mainly involved in catalytic reactions related to carbon dioxide fixation. Genes *LXC004308* and *LXC000032* both encode fructose-1,6-bisphosphatase, which functions to convert fructose-l,6-bisphosphate to fructose-6-phosphate and plays a key role in sugar iso-metabolism and photosynthesis [26]. The gene *LXC003646* encodes phosphoglycerate kinase, an enzyme that is essential for the survival of every living organism and of which the deficiency can cause dysfunctions in the metabolism of the organism [27]. Gene *LXC002690* encodes ribulose-phosphate-3-epimerase, which mainly catalyzes the interconversion of ribulose-5-phosphate and xylulose-5-phosphate in the Calvin cycle, oxidizes pentose, and plays an important role in carbon fixation during photosynthesis [28]. *LXC001520* and *LXC007344* encode genes associated with lysosome synthesis [29].

Table 2 displays basic information about the genes in the key enrichment pathways that had a strong association with strain attributes and a *p*-value < 0.05. The magnitude of their correlation values ranged from 0.68 to 0.87. Two genes with a strong link to strain features were in the oxidative phosphorylation pathway, one was in the fatty acid metabolism pathway, and seven in the porphyrin and chlorophyll metabolism system. These genes may be key to producing differences in strain characteristics between mutants and the control, and their specific functions are shown in Table 2. Genes *LXC005216* and *LXC001791* encode iron chelatase and magnesium chelatase, respectively, which are key enzymes in the catalytic reaction of chlorophyll synthesis [30]. The two genes involved in oxidative phosphorylation (*LXC007989* and *LXC000548*) encode NADH dehydrogenase and ATP synthase subunit d, respectively. NADH dehydrogenase is an enzyme located in the inner mitochondrial membrane that catalyzes the transfer of electrons from NADH to coenzyme Q [31]. ATP synthase subunit d plays an important role in the cytochrome c redox chain. It converts ADP and inorganic phosphate into ATP, which provides the energy required by the cell [32]. The enzymes encoded by these two genes are mainly related to energy metabolism. The gene *LXC005122* encodes enoyl-[acyl-carrier-protein] reductase, catalyzes the final reduction of fatty acids, and is essential for fatty acid synthesis [33].

## 4. Discussions

In this study, WGCNA identified three modules (blue, red, and tan) as having strong association with salinity.

Gene expression levels in the blue and red modules were upregulated in all strains as the salinity increased. KEGG analysis revealed that the most significant enrichment pathway in the blue module was the proteasome, and in the red module, it was photosynthesis-mediated carbon fixation. Proteasome and photosynthetic carbon fixation genes displayed increased expression at 60‰ compared to 45‰ and 30‰ salinity in each sample. In the proteasome pathway, gene expression in the X5 strain was higher than that of the control and X3 strains, whereas the expression of genes in the carbon fixation of the photosynthesis pathway was mostly higher in the control and X3 groups compared to the X5 strain. These results suggest that the three strains target different metabolic pathways in response to high salinity stress, with X5 targeting mostly the proteasome and the other two targeting primarily the carbon fixation of photosynthesis. The former is primarily engaged in protein breakdown, whereas the latter responds to excessive salt stress by increasing photosynthesis. 

In addition to the differences described above, the major pathways enriched in the blue module (i.e., enriched pathways with a larger percentage of genes) included ribosome, RNA transport, and the biosynthesis of secondary metabolites. The presence of ribosome and RNA transport further suggested the possibility that protein-related metabolism may be more active in strain X5 at 60‰ compared to strain X3 and the control. The biosynthesis of secondary metabolites suggested that some of the secondary metabolites may differ more in strain X5 at 60‰ compared to X3 and the control. In the red module, the main enriched pathways (the enriched pathways with significantly different or a larger percentage of genes) were the biosynthesis of secondary metabolites, the biosynthesis of amino acids, and carbon metabolism. The presence of carbon metabolism further suggested that the control and X3 strains have more active carbon metabolism than the X5 strain at 60‰. The presence of the biosynthesis of secondary metabolites and biosynthesis of amino acids suggested that at 60‰, some secondary metabolites and amino acids may also be more different in the X3 and control groups compared to strain X5.

**Figure 10:**
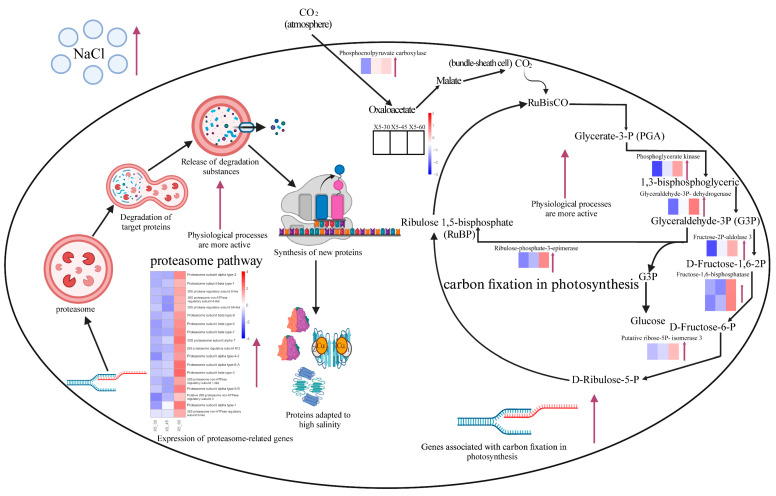
Hypothesis mechanism of X5 in response to high-salinity treatment. Note: The metabolic pathways in the figure are, from left to right, the proteasome pathway and photosynthetic carbon fixation. The heatmap labeled next to the gene names in the figure is the expression of that gene. The heatmap represents samples X5-30, X5-45, and X5-60 from left to right. The black arrows in the figure represent the direction of metabolism. The red arrows indicate that the nearby genes are upregulated or the metabolic pathway is more active.

According to previous studies, proteasomes are involved in the response to abiotic salt stress, and proteasomes function with ubiquitination to reconfigure the organism’s proteome composition in order to make it more adaptable to high-salt environments at the proteomic level [34,35,36,37]. Furthermore, during salt stress, genes associated with the proteasome’s 26S subunit were found to be upregulated in rapeseed (*Brassica napus*) [38]. Therefore, the proteasome may play a major role in salt tolerance, and linkage analysis revealed that *LXC001520* and *LXC007344* were strongly correlated with the proteasome. We hypothesize that the proteasome plays a role in responding to salt stress in the X5 strain by resetting the organism’s proteome composition. This adaptation may help the organism better survive in high-salt environments at the proteomic level (Figure 10). Previous studies reported that salt stress has a significant impact on photosynthesis, thereby affecting algal growth and development [39,40,41,42,43]. As a result, when exposed to stress, boosting gene expression in the photosynthesis-related pathway likely helps algae maintain normal energy and material metabolism, as observed in strain X5 under salt stress (Figure 10). The gene *LXC003712*, which is associated with carbon fixation in photosynthesis and was upregulated in all three strains at the salinity of 60‰, encodes glyceraldehyde-3-phosphate dehydrogenase, a key enzyme that catalyzes the formation of 3-phosphoglyceraldehyde from 1,3-bisphosphoglyceric acid [44,45,46]. The expression of *LXC003712* and the rest of the genes involved in carbon fixation by photosynthesis were also upregulated in strain X5 of 60‰ (Figure 10). According to previous reports, the expression of the genes involved in photosynthetic carbon fixation are also upregulated in higher plants exposed to abiotic stresses (e.g., high salt, high osmotic pressure) that affect photosynthesis [47,48]. This suggested that the red alga *G. lemaneiformis* may have a mode of regulation that is similar to that of higher plants in response to similar stresses. Based on the results of our analyses, we inferred that carbon fixation in strain X5 is activated in response to high salt stress (Figure 10).

Four modules from the WGCNA (black, cyan, light cyan, and yellow) were identified as having a high association with strain traits. According to KEGG analysis, the most significant enrichment pathway for the black module was lipid metabolism, in the cyan module it was oxidative phosphorylation, in the light-cyan module it was SNARE interactions in vesicular transport, and in the yellow module it was porphyrin and chlorophyll metabolism. At 30‰ salinity, strain X5 had a significantly lower expression of the genes involved in porphyrin and chlorophyll metabolism compared to the control and X3 groups. It is clear that the genes that are highly associated with the strains are related mostly to porphyrin and chlorophyll metabolism (Table 2). Porphyrin and chlorophyll metabolism primarily affects photosynthesis, and it plays key roles in energy transmission [49]. The downregulation of genes involved in porphyrin and chlorophyll metabolism (Figure 8d) in strain X5 would obviously impair energy transfer and increase light energy dissipation. This may further explain why among the chlorophyll fluorescence parameters that have been measured in previous studies with the strain [5], the non-photochemical quenching (NPQ) values of strain X5 were higher than those of the control and X3 samples at 30‰. In turn, NPQ is an important parameter when tolerating stress; therefore, strain X5 may show certain stress tolerance characteristics when cultured in normal seawater. *LXC005216*, a gene involved in the porphyrin and chlorophyll metabolic pathways, encodes an iron chelatase [50,51], while *LXC001791* encodes a magnesium chelatase [52]. In the process of chlorophyll production, glutamyl-tRNA first creates protoporphyrins through a series of events. Protoporphyrin produces ferrous hemoglobin and photosensitive pigments in the presence of ferrochelatase and magnesium chelatase, respectively. Ferrous hemoglobin and photosensitive pigments can produce chlorophyll a, chlorophyll b, and other pigments in the following reaction, in which ferrochelatase and magnesium chelatase play critical roles [53]. The downregulation of these two enzymes is expected to inhibit pigment production, which may impact the energy transfer of photosynthesis, thereby increasing light energy dissipation.

At 30‰ salinity, the X5 strain exhibited a significantly lower expression of genes involved in oxidative phosphorylation metabolism compared to the control and X3 groups (Figure 8b). Oxidative phosphorylation metabolism is mainly related to energy metabolism [54], and two genes in this pathway (*LXC007989*, *LXC000548*) correlated well with strain traits (Table 2). *LXC007989* encodes a subunit of NADH dehydrogenase and *LXC000548* encodes ATP synthase subunit d. Both enzymes are related to energy metabolism [55]. In the X5-30 group, the low expression of genes in this pathway (Figure 8b) may have reduced energy metabolism so that life activities would be impaired compared to those in the Ctr-30 and X3-30 groups. At 30‰ salinity, the expression of genes in the SNARE interactions in the vesicular transport pathway was higher in strain X5 compared to the control and X3 groups (Figure 8c). SNARE interactions in vesicular transport are mainly associated with the transport of macromolecules and granular substances across cell membranes [56]. The higher gene expression in the X5-30 sample may alter the transcellular membrane transport of macromolecules and granular substances compared to the Ctr-30 and X3-30 groups. The expression of genes related to the synthesis of secondary metabolites differed greatly between the X5-30 and Ctr samples, except for glutamyl-tRNA reductase, acetyl-CoA carboxylase, and ubiquinone biosynthesis monooxygenase genes (Figure 8a). The enriched pathways were involved in amino acid metabolism, chlorophyll metabolism, lipid metabolism, etc. After running the KEGG analysis for these genes a second time, the most significant enriched pathways were related to lipid metabolism (Figure 9). The differences in the gene expression between X5 and Ctr samples at 30‰ suggested that strain X5 differs from the Ctr strain at this salinity in multiple metabolic pathways, and the differences in lipid metabolism are the most significant.

Our results provide a reference for studies of the resistance of algae to stress. By studying the characteristics of the mutant X5 strain, we identified enriched pathways and genes correlated with the characteristics of the X5 strain. These data can be used to guide the selection of superior strains of *G. lemaneiformis* in the future.

## 5. Conclusions

We found that the most important enrichment mechanisms for salt tolerance in *G. lemaneiformis* may be the proteasomal and photosynthetic carbon fixation. To cope with high salt stress, the mutant X5 may focus on the proteasome pathway, whereas the control strain may focus on photosynthesis and carbon fixation. The high expression of genes in the proteasome pathway in the mutant may be an important reason for the mutant’s tolerance to high salt levels compared to the control. In future work, protein profiling can be performed on the control and mutant before and after high salt treatment to determine the changes in the protein profiles of the control and mutant. 

Among the strain-associated enrichment pathways, the genes of strain X5 cultured at 30‰ salinity were downregulated in the porphyrin and chlorophyll metabolic pathway, which may be linked to the higher NPQ of this strain compared to the others. The differences in gene expression in the X5-30 group in the synthesis of secondary metabolites, oxidative phosphorylation, and SNARE interactions in vesicular transport pathways compared to the control suggest that the X5-30 group may differ from the Ctr-30 group in lipid metabolism, energy metabolism, and vesicular transport. The mutant X5-30 exhibits differences compared to the control Crt-30 in multiple physiological processes. In future work, metabolomic profiling can be performed on X5-30 and Crt-30 to elucidate the physiological differences between the samples at the metabolic level.

## Figures and Tables

**Table 1:** Basic information on genes in proteasomes and carbon fixation in the photosynthetic pathway with a good correlation with salinity.

Gene_Id	Characteristic	Correlation	*p*-Value	Metabolic Pathway	Function
*LXC000032*	Salinity	0.7282	0.0261	Carbon fixation in photosynthetic organisms	Fructose-1,6-bisphosphatase, cytosolic [*Gracilariopsis chorda*]
*LXC001520*	Salinity	0.8079	0.0084	Proteasome	Proteasome subunit β type-1 [*G. chorda*]
*LXC007344*	Salinity	0.6991	0.0361	Proteasome	Proteasome subunit β type-5 [*G. chorda*]
*LXC003712*	Salinity	0.7958	0.0103	Carbon fixation in photosynthetic organisms	Glyceraldehyde-3-phosphate dehydrogenase, chloroplastic [*G. chorda*]
*LXC002690*	Salinity	0.7717	0.0148	Carbon fixation in photosynthetic organisms	hypothetical Ribulose-phosphate 3-epimerase [*G. chorda*]
*LXC003646*	Salinity	0.7152	0.0303	Carbon fixation in photosynthetic organisms	Phosphoglycerate kinase, chloroplastic [*G. chorda*]
*LXC004308*	Salinity	0.7890	0.0114	Carbon fixation in photosynthetic organisms	Fructose-1,6-bisphosphatase, chloroplastic [*G. chorda*]

**Table 2:** Basic information on genes in lipid metabolism, oxidative phosphorylation, and porphyrin and chlorophyll metabolism pathways with a good correlation with strains.

Gene_Id	Characteristic	Correlation	*p*-Value	Metabolic Pathway	Function
*LXC007989*	Strains	−0.79982	0.009656	Oxidative phosphorylation	NADH dehydrogenase [ubiquinone] 1 β subcomplex subunit 10-B [*Gracilariopsis chorda*]
*LXC000548*	Strains	−0.87707	0.001898	Oxidative phosphorylation	ATP synthase subunit d, mitochondrial [*G. chorda*]
*LXC002968*	Strains	−0.79724	0.010072	Porphyrin and chlorophyll metabolism	Glutamate-1-semialdehyde 2,1-aminomutase [*G. chorda*]
*LXC000696*	Strains	−0.85331	0.003438	Porphyrin and chlorophyll metabolism	Geranylgeranyl diphosphate reductase, chloroplastic [*G. chorda*]
*LXC005913*	Strains	−0.80719	0.008533	Porphyrin and chlorophyll metabolism	Delta-aminolevulinic acid dehydratase, chloroplastic [*G. chorda*]
*LXC003053*	Strains	−0.88065	0.001717	Porphyrin and chlorophyll metabolism	Uroporphyrinogen decarboxylase [*G. chorda*]
*LXC005216*	Strains	−0.77977	0.013206	Porphyrin and chlorophyll metabolism	Ferrochelatase [*G. chorda*]
*LXC001791*	Strains	−0.72984	0.025602	Porphyrin and chlorophyll metabolism	Magnesium–chelatase subunit ChlH, chloroplastic [*G. chorda*]
*LXC005122*	Strains	−0.71535	0.030264	Fatty acid biosynthesis	Enoyl-[acyl-carrier-protein] reductase [NADH] FabI [*G. chorda*]
*LXC004426*	Strains	−0.68286	0.042645	Porphyrin and chlorophyll metabolism	Glutamate–tRNA ligase, chloroplastic/mitochondrial [*G. chorda*]

## Data Availability

The methods, materials, and data used in this study are fully delineated in the article or Supplementary Material. The raw data supporting the conclusions of this article will be made available by the authors on request.

## Supplementary Materials

The following supporting information can be downloaded at: https://www.mdpi.com/article/10.3390/genes15060781/s1, Table S1: Statistical analysis of transcriptome sequencing quality.
