# Supplementary material for: Analysis of Hyperosmotic Tolerance Mechanisms in Gracilariopsis lemaneiformis Based on Weighted Co-Expression Network Analysis
Source: Genes (Basel). 2024 Jun 13;15(6):781. doi: 10.3390/genes15060781 (PMC11203144; doi:10.3390/genes15060781)
Supplement: Supplementary file 1 [file genes-15-00781-s001.zip › genes-3028151-supplementary.pdf]

Title: Analysis of Hyperosmotic Tolerance Mechanisms in *Gracilariopsis lemaneiformis* Based on Weighted Co-Expression Network Analysis

Authors: Baoheng Xiao, Xiaoqing Feng, Pingping Li, Zhenghong Sui*

Institute: Key Laboratory of Marine Genetics and Breeding, Ocean University of China, Ministry of Education, Qingdao 266003, China.

The first author: Baoheng Xiao E-mail: klad1990@163.com

Co-author: Xiaoqing Feng E-mail: 17086260662@163.com

Co-author: Pingping Li E-mail: lpp0780@163.com

***Author for Correspondence:** Zhenghong Sui, Phone: (+86) 532-82031128, E-mail: suizhengh@ouc.edu.cn

Table S1. Statistical analysis of transcriptome sequencing quality

Sample	Clean_reads	Clean_bases	Error_rate	GC_pct	Total_map	Unique_map	Multi_map
Ctr_30_1	40732452	6.11G	0.03%	52.55%	94.53%	92.75%	1.78%
Ctr_30_2	43366036	6.50G	0.03%	52.54%	94.99%	93.18%	1.81%
Ctr_30_3	46188882	6.93G	0.03%	52.31%	95.70%	93.64%	2.06%
Ctr_45_1	44763648	6.71G	0.02%	52.53%	95.48%	93.70%	1.78%
Ctr_45_2	44430048	6.66G	0.02%	52.64%	96.33%	94.53%	1.80%
Ctr_45_3	41090130	6.16G	0.03%	52.36%	96.56%	94.82%	1.74%
Ctr_60_1	42960932	6.44G	0.03%	51.72%	91.18%	89.98%	1.20%
Ctr_60_2	39963268	5.99G	0.03%	50.32%	79.52%	78.42%	1.10%
Ctr_60_3	44980210	6.75G	0.03%	51.10%	88.67%	87.27%	1.40%
X3_30_1	41017342	6.15G	0.03%	52.62%	93.03%	90.41%	2.62%
X3_30_2	47027778	7.05G	0.03%	52.55%	93.05%	90.50%	2.55%
X3_30_3	41918254	6.29G	0.03%	52.49%	91.26%	88.61%	2.65%
X3_45_1	43149424	6.47G	0.03%	52.73%	94.72%	92.79%	1.93%
X3_45_2	46002638	6.90G	0.03%	52.66%	94.01%	91.86%	2.16%
X3_45_3	45645662	6.85G	0.03%	52.64%	95.31%	93.02%	2.29%
X3_60_1	40185038	6.03G	0.03%	50.12%	71.32%	69.89%	1.43%
X3_60_2	42965412	6.44G	0.03%	52.07%	72.27%	71.01%	1.26%
X3_60_3	43357058	6.50G	0.03%	51.55%	86.84%	85.15%	1.69%
X5_30_1	45609248	6.84G	0.02%	51.35%	71.49%	69.77%	1.72%
X5_30_2	44626012	6.69G	0.02%	51.95%	91.92%	89.48%	2.44%
X5_30_3	46744660	7.01G	0.02%	51.10%	78.20%	76.17%	2.02%
X5_45_1	42653624	6.40G	0.02%	51.99%	95.83%	93.08%	2.75%
X5_45_2	44468940	6.67G	0.03%	52.09%	96.35%	94.16%	2.19%
X5_45_3	43296338	6.49G	0.02%	51.76%	95.20%	92.23%	2.97%
X5_60_1	48671912	7.30G	0.03%	51.76%	75.70%	74.40%	1.30%
X5_60_2	45112380	6.77G	0.03%	51.59%	77.11%	75.86%	1.25%
X5_60_3	43875134	6.58G	0.03%	49.81%	64.23%	63.10%	1.14%

Note: Sample: name of the sample.

Clean_reads: number of reads after filtering the raw data.

Clean_bases: number of bases filtered from raw data.

Error_rate: overall sequencing error rate of the data.

GC_pct: percentage of G and C in clean reads for each of the four bases.

Total_map: number and percentage of reads matched to the genome.

Unique_map: number and percentage of reads aligned to a unique position in

the reference genome (for subsequent quantitative data analysis of reads)

Multi_map: number and percentage of reads aligned to multiple positions in the reference genome.
